# Juvenile Oligoarthritis und Uveitis: Bleibt es dabei?

**DOI:** 10.1007/s00393-026-01890-6

**Published:** 2026-10-30

**Authors:** Andreas Wörner, Charlotte Wortmann, Arnd Heiligenhaus

**Affiliations:** 1 Universitäts-Kinderspital beider Basel, Universität Basel, Spitalstrasse 33, 4031 Basel, Schweiz; 2 Augenzentrum am St. Franziskus Hospital, Hohenzollernring 74, 48145 Münster, Deutschland; 3 Universitätsklinikum Duisburg-Essen, Essen, Deutschland

**Keywords:** Juvenile idiopathische Arthritis, Kinderrheumatologie, Transition, Uveitis, Okuläre Komplikationen, Juvenile idiopathic arthritis, Pediatric rheumatology, Transition, Uveitis, Ocular complications

## Abstract

Die juvenile idiopathische Arthritis und die damit assoziierte Uveitis persistieren nicht selten bis in das Erwachsenenalter. Im Beitrag werden Klinik und Verlauf von oligoartikulärer juveniler idiopathischer Arthritis und assoziierter Uveitis im Kindes- und Jugend- sowie Erwachsenenalter dargelegt. Die besonderen Herausforderungen in Diagnostik und Therapie an der Schnittstelle zur Rheumatologie im Erwachsenenalter (Transition) werden diskutiert.

## Abstract (en)

Juvenile idiopathic arthritis and associated uveitis may persist into adulthood. This article addresses the clinical aspects and trajectory of oligoarticular juvenile idiopathic arthritis and associated uveitis in the pediatric and young adult age. Specific challenges in diagnosis and treatment at the interface between pediatric and adult rheumatology (transition) are discussed.

Die juvenile idiopathische Arthritis (JIA) ist definiert als eine Arthritis unbekannter Ursache in einem oder mehreren Gelenken, die länger als 6 Wochen besteht, vor dem 16. Geburtstag beginnt und bei der andere Ursachen einer Arthritis ausgeschlossen sind. Die klinische Präsentation der JIA ist heterogen. Die International League of Associations for Rheumatology (ILAR) teilt die JIA in 6 Kategorien und einen undifferenzierten Subtyp ein [1], welche die klinischen Zeichen differenzieren, Laborwerte wie Rheumafaktor, antinukleäre Antikörper (ANA) und HLA-B27 mit einschließen und den Krankheitsverlauf berücksichtigen.

Die oligoartikuläre JIA (oJIA) ist der häufigste Subtyp. In den ersten 6 Monaten der Erkrankung besteht eine Arthritis in maximal 4 Gelenken (persistierende oJIA); sind nach den ersten 6 Monaten zusätzliche Gelenke betroffen, bezeichnet man sie als extended oJIA. Bei mehr als zwei Drittel der Kinder in dieser Subgruppe zeigen sich positive antinukleäre Antikörper (ANA).

## Klinik und Verlauf der oJIA

Bei der oJIA sind häufig mittelgroße und große Gelenke wie Knie‑, Sprung‑, Hand- oder Ellenbogengelenke, selten kleinere Gelenke an Händen und Füßen betroffen. Eine Symmetrie betroffener Gelenke ist seltener als bei anderen JIA-Subtypen. Gelenkschmerzen sind nicht immer eines der vorrangigen Symptome, und abhängig vom Alter des Kindes und der Beobachtung der Eltern kann eine verkürzte Belastung einer betroffenen Extremität eines der ersten Zeichen sein. Eine Morgensteifigkeit kann bereits bei Kleinkindern vorhanden sein.

Eine Besonderheit der juvenilen Arthritispräsentation ist die mögliche Beteiligung der Kiefergelenke, welche sich typischerweise durch eingeschränkte oder asymmetrische Kieferöffnung zeigt. Aufgrund der initial meist fehlenden Schmerzen ist die klinische Diagnose einer Kiefergelenkarthritis nicht immer einfach und benötigt häufig eine ergänzende MRT-Bildgebung.

Die JIA-assoziierte systemische Entzündung kann Auswirkungen auf weitere Organsysteme während des Wachstumsalters zeigen

Der Altersgipfel der oJIA bei Erstmanifestation liegt zwischen 2 und 4 Jahren. Mädchen sind häufiger betroffen als Jungen. Eine JIA bedeutet eine systemische Entzündung in einem wachsenden Individuum, was insbesondere in dieser jungen Altersgruppe Folgen hat, nicht nur für die Gelenke, sondern auch für das weitere muskuloskeletale und hormonale Organsystem. Unbehandelt werden innerhalb weniger Wochen entlastungsbedingte Muskelhypotrophien sichtbar, ossäre Wachstumsstörungen mit Skelettasymmetrien, wie z. B. eine Beinlängendifferenz mit Beckenschiefstand können bereits nach wenigen Monaten bestehen. Die systemische Entzündung kann ohne ausreichende Behandlung Auswirkungen auf weitere Organsysteme haben und u. a. die Körperendlänge negativ beeinflussen.

Die Diagnosestellung wird nicht selten durch einen schleichenden Symptombeginn verzögert, über verschiedene Länder hinweg beträgt sie etwa 3 bis 4 Monate [2, 3]. Aufgrund der breiten Differenzialdiagnose sollte diese in einem kinderrheumatologischen Zentrum erfolgen [4]. Eine mögliche Verzögerung ist von zentraler Bedeutung für die Diagnose einer JIA-assoziierten Uveitis, da die Augenuntersuchung in den meisten Fällen erst nach Diagnose der JIA erfolgt. Daher ist eine zeitnahe ophthalmologische Beurteilung indiziert. Nach den aktuellen Leitlinien sollte diese innerhalb von 2 Wochen erfolgen, da der Nachweis einer Uveitis das therapeutische Procedere ändert [5, 6].

Der klinische Verlauf der oJIA ist günstiger als in anderen JIA-Kategorien. Während Langzeitstudien eine klinische Remission ohne Medikamente bei etwa einem Drittel aller JIA-Diagnosen dokumentiert haben, liegt sie bei persistierender oJIA bei etwa 60 % [7]. Bei Vorliegen einer „extended“ oJIA ist die Rate der therapiefreien Remission jedoch mit unter 35 % deutlich niedriger [8].

Die Verhinderung von Langzeitkomplikationen ist ein zentrales Ziel der Behandlung

Die Verhinderung von Langzeitkomplikationen ist ein zentrales Ziel der Behandlung. Im Kindes- und Jugendalter kommt neben entzündungsbedingten osteoartikulären Schäden zusätzlich das Risiko wachstumsbedingter Auswirkungen auf betroffene Gelenke und Extremitäten hinzu. Die Rate arthritisbedingter Folgeschäden im jungen Erwachsenalter variiert in Studien und wird mit 3–25 % bei JIA-Diagnose im Kindesalter angegeben [9, 10]. Die Länge der Krankheitsdauer im Kindesalter, Einnahme von systemischen Kortikosteroiden und später begonnener Einsatz von Biologika sind Einflussfaktoren [11]. Krankheitsbedingte Einschränkungen, welche sich auf die Lebensqualität allgemein auswirken, bestehen je nach Langzeitstudie bei 30 bis knapp 50 % [12–14]. Das Risiko von Komorbiditäten ist erhöht; Registerdaten für JIA zeigen bereits im jungen Erwachsenenalter eine höhere Rate weiterer Autoimmunerkrankungen wie Schilddrüsenfunktionsstörungen oder chronisch-entzündliche Darmerkrankung sowie kardiovaskuläre Risikofaktoren (arterielle Hypertension, Adipositas, Dyslipidämie), Angststörungen und Depression [15, 16], wenngleich die Komorbiditätsinzidenz bei oJIA niedriger als in anderen JIA-Subtypen angegeben wird. Damit können bei erfolgter Transition ins Erwachsenenalter bei einem nicht unerheblichen Teil der jungen Patienten artikuläre und Lebensqualitätseinschränkungen vorliegen, welche in der weiteren Therapie berücksichtigt werden müssen.

## JIA-assoziierte Uveitis

Von den Patienten mit oJIA entwickeln 9–13 % eine Uveitis, wobei Patienten mit persistierender (15–20 %) und extended oJIA (20–40 %) besonders häufig betroffen sind. Besonders häufig (bis zu 30 %) ist die Uveitis bei oJIA-Kindern mit frühem Arthritisbeginn und positiven ANA [17]. Oft beginnt die Uveitis unmittelbar mit der Arthritis oder in den ersten Monaten bis wenigen Jahren danach ([18]; Tab. 1).

**Tab. 1:** Empfehlung Uveitisscreening im Kindes- und Jugendalter

JIA-Subgruppe	ANA	Erkrankungsalter (Jahre)	Krankheitsdauer (Jahre)	Empfohlene Frequenz von Spaltlampenuntersuchungen (in Monaten) bei Kindern ohne bereits diagnostizierte Uveitis
Oligoarthritis. RF-negative Polyarthritis, Psoriasisarthritis, unklassifizierte Arthritis	+	≤ 6	≤ 4	3
	+	≤ 6	> 4	6
	+	≤ 6	≥ 7	12
	+	> 6	≤ 2	6
	+	> 6	> 2	12
	–	≤ 6	≤ 4	6
	–	≤ 6	> 4	12
	–	> 6	n.r.	12
Enthesitis-assoziierte Arthritis	n.r.	n.r.	n.r.	3
RF-positive Polyarthritis, systemische Arthritis	n.r.	n.r.	n.r.	12
Patienten mit bereits diagnostizierter Uveitis	n.r.	n.r.	n.r.	Entsprechend dem individuellen Uveitisverlauf, mindestens 3 monatlich

*ANA* antinukleäre Antikörper, *JIA* juvenile idiopathische Arthritis, *RF* Rheumafaktor, *n.r.* nicht relevant

Die JIA-assoziierte Uveitis (JIAU) manifestiert sich nahezu ausschließlich als anteriore Uveitis entsprechend der Standardization of Uveitis Nomenclature (SUN) Working Group [19, 20]. Infolge der Entzündung können die benachbarten Strukturen (Hornhaut, Linse, Trabekelwerk) und auch die Makula in Mitleidenschaft gezogen werden, sofern keine frühzeitige ophthalmologische Diagnose und konsequente antiinflammatorische Therapie erfolgen [21]. Bei der JIAU handelt es sich im typischen Fall um eine chronische, nichtgranulomatöse Uveitis. Die pathophysiologische Grundlage ist ein immunvermittelter Zusammenbruch der Blut-Kammerwasser-Schranke [21]. Mit der Spaltlampe sind dann Vorderkammerzellen und ein erhöhter Einweißgehalt im Kammerwasser (sog. Tyndall-Effekt) erkennbar, der mit der LaserFlare-Messung quantifiziert werden kann [22, 23]. Weitere obligate Untersuchungen dienen der Beurteilung von Visus, Tensio und Fundus.

Das äußere Erscheinungsbild des Auges bleibt oft unauffällig

Von zentraler klinischer Bedeutung ist, dass das äußere Erscheinungsbild des Auges bei der typischen JIA-Uveitis oft unauffällig bleibt. Typische sekundäre Folgen sind hintere Synechien (mit konsekutiver Pupillenverziehung), Bandkeratopathie der Hornhaut, Katarakt, Augendruckerhöhung und Glaukom sowie weiterhin Makulaödem [24–26].

## Welche Verläufe sehen wir im Erwachsenenalter – und was muss die Erwachsenenrheumatologie wissen?

Auch im Erwachsenenalter weist die JIAU typischerweise einen chronischen, weitgehend asymptomatischen Verlauf am äußerlich unauffälligen Auge aus. Weder okulare Schmerzen noch eine Augenrötung sind regelhaft vorhanden, sodass Aktivität und Schweregrad der Uveitis in der Regel erst im Rahmen ophthalmologischer Untersuchungen diagnostiziert werden [27, 28].

Oft ist der Visus initial oder bei niedrigem Entzündungsgrad nicht reduziert. Eine subjektive Visusminderung kann bei höhergradiger Entzündungsaktivität oder infolge sekundärer okulärer Komplikationen auftreten. Nach derzeitigem Kenntnisstand bleibt dieses asymptomatische Erscheinungsbild auch bei einer Persistenz der Uveitis ins Erwachsenenalter erhalten [29, 30].

In der Patientenkohorte in unserem tertiären Uveitiszentrum in Münster wurden etwa zwei Drittel der Patienten auch nach Vollendung des 18. Lebensjahres weiterhin ophthalmologisch betreut. Etwa ein Drittel aller JIAU-Patienten zeigte eine persistierende Uveitisaktivität ins Erwachsenenalter, sodass die augenärztliche Betreuung kontinuierlich fortgesetzt werden muss. Demgegenüber beendete etwa ein Drittel der Patienten die nachfolgenden Kontrollen bei uns, was auf Wechsel von Arbeitsplatz (z. B. Studium) und Wohnort beruhen kann. Auch nach Erzielen therapiefreier Remission sollten regelmäßige augenärztliche Kontrollen (z. B. halbjährlich bis jährlich) zum Nachweis etwaiger Reaktivierung oder sekundärer Komplikationen (z. B. Augendruckanstieg) erfolgen. Die Nachsorge und Kontrollintervalle müssen individuell an den bisherigen Krankheitsverlauf, die Entzündungsaktivität und etwaigen okularen Komplikationen sowie die aktuelle Therapie mit krankheitsmodifizierenden Antirheumatika (DMARD) angepasst werden. Entsprechend den Empfehlungen im Kindesalter sollten 3–12 monatliche Kontrollen fortgesetzt werden. (S2k AWMF Leitlinie Diagnostik und antientzündliche Therapie der Uveitis bei JIA). Patienten mit persistierender Uveitisaktivität sollten auch im Erwachsenenalter regelmäßig und engmaschig in einem auf Uveitis spezialisierten Zentrum augenärztlich überwacht werden.

Die Häufigkeit der notwendigen Verlaufskontrollen richtet sich zusätzlich nach dem Vorliegen entzündungsbedingter okulärer Komplikationen sowie der jeweiligen Therapie. Unter systemischer DMARD-Behandlung steht oft die Kontrolle von Entzündungsaktivität und damit verbundenen Komplikationen im Vordergrund. Eine lokale Steroidtherapie erfordert aufgrund des Risikos einer steroidinduzierten okularen Hypertension regelmäßige Augeninnendruckmessungen. Da bei dem mit JIAU verbundenen Glaukom starke tageszeitliche Druckschwankungen typisch sind, müssen entsprechend über den Tag verteilte Augendruckmessungen (sog. Tagesdruckprofile) dringend empfohlen werden. Bei längerfristiger Entzündungsfreiheit kann das Kontrollintervall auf 3–6 monatliche Intervalle gestreckt werden, während bei hoher Aktivität auch bis zu tägliche/zweitägliche Kontrollen notwendig sein können [18].

Während bei etwa 10 % der JIA-Patienten die Uveitis bereits vor Diagnose der Arthritis besteht, kann sie auch erst mehr als 10 Jahre nach Diagnose einer JIA auftreten. Je nach Alter bei Erstdiagnose der JIA kann das Auftreten einer Uveitis erst im Erwachsenenalter liegen, was aber recht selten ist.

## Therapie

Die Beteiligung der beiden Organsysteme Gelenke und Augen kann in der Behandlung herausfordernd sein. Nicht nur sind die Kinder im Vergleich zu anderen JIA-Formen oft früh betroffen, was ein Risikofaktor für Komplikationen darstellt; die interdisziplinäre Betreuung zwischen Ophthalmologie und Kinder- und Jugendrheumatologie erfordert ein abgestimmtes Vorgehen, um Langzeitrisiken zu minimieren.

Nach Diagnosestellung von oJIA und JIAU steht eine wirksame und sichere Therapie im Vordergrund, mit der Komplikationen vermieden werden können. Seit Mitte der 2010er-Jahre hat sich die Treat-to-target(T2T)-Strategie zur Behandlung der JIA durchgesetzt [31]. Dabei wird eine inaktive Erkrankung als Behandlungsziel angestrebt, deren Kriterien 2011 von Wallace et al. definiert wurden [32]. Hierzu gehören (1) keine aktiven Gelenke, (2) kein Fieber, Exanthem, Serositis, Splenomegalie, Lymphadenopathie in Zusammenhang mit JIA, (3) keine aktive Uveitis entsprechend SUN-Kriterien, (4) Blutsenkungsgeschwindigkeit (BSG) oder C‑reaktives Protein (CRP) normal in Zusammenhang mit JIA, (5) Einschätzung der Krankheitsaktivität durch den Arzt mit dem bestmöglichen Wert auf der verwendeten Skala, und (6) Morgensteifigkeit von weniger als 15 min. Diese Definition leitet das interdisziplinäre Team von Rheumatologen und Ophthalmologen, die Therapie abzustimmen und zu optimieren sowie ein fachdisziplinübergreifendes Behandlungsziel zu formulieren. Die Definition wurde aus einem pädiatrischen Kriterienset entwickelt; dieses wird in Studien jedoch auch nach Erreichen des Erwachsenenalters angewendet, da keine auf die Entzündungsaktivität von sowohl Gelenken als auch Augen bezogene Definition bei Erwachsenen existiert [11].

Bei der oJIA, welche sich im Erwachsenenalter anhaltend präsentiert, gelingt die überlappende Klassifikation einer chronischen Arthritis des Erwachsenenalters nur in 20–60 % der Fälle [11, 33]. Das Vorhandensein einer JIAU ist dabei eines der Kriterien, welches zur Unklassifizierbarkeit führen kann. Die Uveitis gehört daher zu einer der Spezifika der oJIA auch im Erwachsenenalter, und die Diagnose einer JIA bzw. JIAU sollte in dieser Hinsicht auch jenseits des 18. Lebensjahres fortgeführt werden.

Zum Erreichen der T2T-Ziele sind ein frühzeitiger Einsatz von DMARD, eine regelmäßige standardisierte Messung der Krankheitsaktivität sowie eine Therapieanpassung bei nicht ausreichendem Ansprechen erforderlich [34]. Das Erreichen einer inaktiven Erkrankung ist die Voraussetzung für eine klinische Remission (definiert als eine anhaltende inaktive Erkrankung für mehr als 6 Monate), welche eine Vermeidung (weiterer) struktureller Gewebsschäden von Gelenken und Augen ermöglichen kann. Dabei soll eine mindestens 50 %ige Verbesserung der Krankheitsaktivität innerhalb von 3 Monaten und eine als akzeptabel klassifizierte Krankheitsaktivität der oJIA innerhalb von 6 Monaten erreicht werden [4, 6]. Bei Nichterreichen dieses Ziels hat der frühzeitige Einsatz von biologischen DMARD ein besseres Langzeit-Outcome gezeigt [35]. Dieses *Window of opportunity* betrifft vermutlich auch die JIAU. Besonders bedeutsam ist allerdings der oftmals dissoziierte Krankheitsverlauf von Uveitis und Arthritis, sodass alle Therapieentscheidungen interdisziplinär abgestimmt werden sollten.

Die medikamentösen Therapieoptionen sind in Tab. 2 zusammengefasst. Eine besondere Herausforderung für Behandlungsteam und Betroffene ist dabei, dass nur ein kleiner Teil der im klinischen Alltag eingesetzten Medikamente für die oJIA und/oder die JIAU zugelassen ist. Neben partieller altersabhängiger Zulassung für nichtsteroidale Antirheumatika (NSA)R, Triamcinolon hexacetonid und dem Tumornekrosefaktor(TNF)-Inhibitor Adalimumab für die Indikation der chronischen nichtinfektiösen Uveitis ab dem Alter von 2 Jahren sind alle weiteren verwendeten Lokaltherapeutika und DMARD im Geltungsbereich der European Medicines Agency EMA als *off-label* klassifiziert. Die aktualisierten Behandlungsempfehlungen der ProKind Rheumainitiative der Gesellschaft für Kinder- und Jugendrheumatologie GKJR für oJIA [6] berücksichtigen die Zulassungskriterien, weisen jedoch auf die Notwendigkeit von Off-label-Medikation bei Nichterreichen des Therapieziels hin.

**Tab. 2:** Medikamente zur Therapie der oligoartikuläre juvenilen idiopathischen Arthritis (oJIA) und JIA-assoziierten Uveitis im Erwachsenenalter im Geltungsbereich der European Medicines Agency (EMA)

Medikamentengruppe	Wirkstoff	Zulassung oJIA	Zulassung pädiatrische Uveitis	Dosierungsempfehlung	Zulassung im Erwachsenenalter (oJIA oder Uveitis/andere Indikationen)
Lokaltherapie	Prednisolon 1 %/Dexamethason 0,1 % Augentropfen	–	Nein	Entsprechend Aktivität	Ja/andere Entzündungen der vorderen Augenabschnitte
	Triamcinolonhexacetonid	Ja	Nein	0,5–1 mg/kgKG intraartikulär (max. 40 mg/20 mg/2 mg große/mittelgroße/kleine Gelenke)	Ja/off-label bei Uveitis (20–40 mg)
NSAR	Ibuprofen	Ja, > 6 Monate	Nein	30–40 mg/kgKG in 3–4 ED	Ja/keine Wirksamkeit bei Uveitis
	Naproxen	Ja, > 1 Jahr	Nein	10–15 mg/kgKG in 2 ED	Ja/keine Wirksamkeit bei Uveitis
	Indometacin	Ja, > 2 Jahre	Nein	2–3 mg/kgKG in 3–4 ED	Ja/keine Wirksamkeit bei Uveitis
	Diclofenac	Ja, > 9 Jahre	Nein	2–3 mg/kgKG in 2–3 ED	Ja/keine Wirksamkeit bei Uveitis
	Meloxicam	Nein	Nein	0,25–0,375 mg/kgKG in 1 ED	Nein, keine Wirksamkeit bei Uveitis/ Rheumatoide Arthritis, ankylosierende Spondylitis, Arthrose > 16 Jahre
	Celecoxib	Nein	Nein	6–12 mg/kgKG in 2 ED	Nein/keine Wirksamkeit bei Uveitis
csDMARD	Methotrexat	Nein	Nein	9–15 mg/m^2^/Woche p.o. oder s.c.	Nein, off-label bei oJIA und Uveitis/polyartikuläre JIA ab 2 Jahren, rheumatoide Arthritis, Psoriasis vulgaris, Morbus Crohn
bDMARD	Adalimumab	Nein	Ja	20 mg/2 Wochen < 30 kg, 40 mg/2 Wochen > 30 kg	Nein, off-label bei oJIA/polyartikuläre JIA ab 2 Jahren, Enthesitis-assoziierte Arthritis ab 6 Jahren, nichtinfektiöse intermediäre, posteriore oder Panuveitis, rheumatoide Arthritis, Psoriasisarthritis, ankylosierende Spondylitis, nichtradiographischer Spondyloarthritis, Morbus Crohn, Colitis ulcerosa, Hidradenitis suppurativa
	Infliximab	Nein	Nein	3–5 mg/kgKG/4–8Wochen^1, 2^ intravenös	Nein, off-label bei oJIA und Uveitis/polyartikulärer JIA ab 2 Jahren, rheumatoider Arthritis, Psoriasisarthritis, ankylosierender Spondylitis, Morbus Crohn, Colitis ulcerosa, Plaque-Psoriasis
	Golimumab	Nein	Nein	30 mg/m^2^KOF/Monat, 50 mg/Monat, abweichend bei chronisch-entzündlichen Darmerkrankungen^2^	Nein, off-label bei oJIA/polyartikulärer JIA ab 2 Jahren, rheumatoider Arthritis, Psoriasisarthritis, ankylosierender Spondylitis, nichtradiographischer axialer Spondyloarthritis, Colitis ulcerosa
	Certolizumab pegol	Nein	Nein	200 mg/2 Wochen oder 400 mg/4 Wochen^2^	Nein, off-label bei oJIA/rheumatoider Arthritis, axialer Spondyloarthritis, Psoriasisarthritis, Plaque-Psoriasis
	Tocilizumab	Nein	Nein	8–12 mg/kgKG/4 Wochen intravenös^1,3^ oder 162 mg/1–3 Wochen subkutan^1^	Nein, off-label bei oJIA und Uveitis/polyartikulärer JIA ab 2 Jahren und systemischer JIA ab 1 Jahr, rheumatoider Arthritis, Riesenzellarteriitis, Zytokinfreisetzungssyndrom, systemische Sklerose-assoziierter interstitieller Lungenerkrankung
	Abatacept	Nein	Nein	250–500 mg (Kinder)^3^, < 60 kgKG 500 mg, > 60 kgKG 750 mg, > 100 kgKG 1 g (Erwachsene)/4 Wochen intravenös oder 50–125 mg/Woche subkutan^1,3^	Nein, off-label bei oJIA und Uveitis/polyartikulärer JIA ab 2 Jahren, rheumatoider Arthritis
	Rituximab	Nein	Nein	375 mg/m^2^KOF/Woche für 4 Wochen, danach 500 mg nach 6, 12, 18 Monaten oder 1000 mg im Abstand von 2 Wochen alle 6 Monate^1^	Nein, off-label bei Uveitis/rheumatoider Arthritis, ANCA-assoziierter Vaskulitis, Pemphigus vulgaris, Non-Hodgkin-Lymphom
tsDMARD	Baricitinib	Nein	Nein	2–4 mg/Tag^1,3^	Nein, off-label bei oJIA und Uveitis/polyartikulärer JIA, Enthesitis-assoziierter JIA, juveniler Psoriasisarthritis ab 2 Jahren, rheumatoider Arthritis, atopischer Dermatitis, Alopecia areata
	Tofacitinib	Nein	Nein	2 × 3–5 mg/Tag^1,3^, abweichend bei chronisch-entzündlichen Darmerkrankungen^2^	Nein, off-label bei oJIA und Uveitis/polyartikulärer JIA, juveniler Psoriasisarthritis ab 2 Jahren, rheumatoider Arthritis, Psoriasisarthritis, ankylosierender Spondylitis, Colitis ulcerosa
	Upadacitinib	Nein	Nein	3–6 mg 2×/Tag (< 12 Jahre)^3,4^, 15 mg retard 1×/Tag (> 12 Jahre)^1,3^, abweichend bei chronisch-entzündlichen Darmerkrankungen^2^	Nein, off-label bei oJIA und Uveitis/atopischer Dermatitis, Alopecia areata und Vitiligo ab 12 Jahren, Psoriasisarthritis, rheumatoider Arthritis, Psoriasisarthritis, ankylosierender Spondylitis, nichtradiographischer Spondyloarthritis, Riesenzellarteriitis, Colitis ulcerosa, Morbus Crohn

*ANCA* antineutrophile zytoplasmatische Antikörper, * NSAR* nichtsteroidale Antirheumatika, *csDMARD* conventional synthetic disease-modifying antirheumatic drug (DMARD), *bDMARD* biologic DMARD, *tsDMARD* targeted synthetic DMARD

^1^ abhängig von der Indikation

^2^ Aufdosierungsschema zu Behandlungsbeginn

^3^ gewichtsabhängig

^4^ Zulassung bisher nur im Geltungsbereich der US Food and Drug Administration (FDA) für polyartikuläre JIA und Psoriasis-assoziierte JIA ab 2 Jahren

## Transition bei JIA und assoziierter Uveitis

Transition bezeichnet einen Prozess der medizinischen Betreuung, welcher im Jugendalter in der Pädiatrie beginnt und sich nach der Übergabe an die Erwachsenenmedizin bis ins junge Erwachsenenalter erstreckt.

Während die Prognose der artikulären Verläufe bei der oJIA im Vergleich zu anderen Formen der JIA günstiger ist und mehr als die Hälfte der betroffenen Kinder und Jugendlichen im Erwachsenenalter keine Aktivitätszeichen mehr aufweisen (Minden et al. 2012), ist die JIAU eine prognoserelevante Begleiterkrankung.

40–60 % der Jugendlichen benötigen eine Weiterbetreuung in der Erwachsenenmedizin

Bei Auftreten einer oJIA und einer assoziierten Uveitis benötigen 40–60 % der Jugendlichen eine Weiterbetreuung in der Erwachsenenmedizin. Wenn sich abzeichnet, dass die betroffene jugendliche Person und das Behandlungsteam mit einer ins Erwachsenenalter andauernden Erkrankungsaktivität rechnen müssen, sollte ab dem Alter von 12–14 Jahren ein Transitionsprozess begonnen werden.

Eine Strukturierung der Transition ist dabei essenziell, um eine reibungslose Weiterbetreuung zu gewährleisten; die European League Against Rheumatism (EULAR) und die Pediatric Rheumatology European Society (PRES) haben hierzu Empfehlungen erarbeitet [36].

Untersuchungen zeigen, dass ein fehlendes Transitionskonzept zu einer erhöhten Rate von „loss to follow-up“, erhöhter Krankheitsaktivität und geringerer Lebensqualität im jungen Erwachsenenalter führen [37]. Gleichzeitig können je nach lokaler Versorgungsstruktur zusätzliche Barrieren in Form von fehlenden Ressourcen in Bezug auf Finanzierung, Personal und Ausbildung bestehen, welche die Umsetzung der geltenden Empfehlungen erschweren [38].

Der Wechsel von kinder- in die erwachsenenzentrierte rheumatologische Betreuung bedeutet in den meisten Fällen einen Wechsel des Behandlungsteams. Der Aufbau eines lokalen Netzwerks von Kinder- und Erwachsenenrheumatologen ist hierbei essenziell. Ausbildungssituation und möglicher Wohnortwechsel aufgrund Ausbildung oder Studium spielen bei der Auswahl der Transitionspartner eine wichtige Rolle. Die GKJR hat Unterlagen für Kinderrheumatologie, internistische Rheumatologie und Jugendliche bzw. junge Erwachsene erarbeitet, welchen einen reibungslosen Übergang erleichtern sollen (Transitionsunterlagen GKJR, https://www.gkjr.de/fur-aerztinnen-und-aerzte/transitionsunterlagen/).

Mit dem Übergang der JIAU vom Kindes- ins Erwachsenenalter ist kein obligater Wechsel des Augenarztes verbunden, da die ophthalmologische Versorgung altersunabhängig durch Fachärzte für Augenheilkunde erfolgt. Dennoch ergeben sich in diesem Alter häufig Veränderungen der Lebenssituation, beispielsweise durch den Beginn einer Berufsausbildung oder eines Studiums, die zum Wechsel von Wohnort und Augenarzt führen können. Regionaler Ärztemangel kann diesbezüglich zu einer Versorgungslücke führen. Ursachen hierfür sind die begrenzte Zahl der auf Uveitis spezialisierten Zentren sowie die eingeschränkten Kapazitäten vieler ophthalmologischer Praxen und Kliniken, die häufig keine neuen Patienten mehr aufnehmen können. Dieser Versorgungsengpass stellt eine relevante Herausforderung für die kontinuierliche Betreuung von Patienten mit chronischer JIAU im Erwachsenenalter dar und birgt das Risiko von Versorgungslücken während der Transition.

Für die Sicherstellung einer kontinuierlichen spezialisierten Versorgung kann es hilfreich sein, Patienten an Augenärzte zu vermitteln, die über eine besondere Expertise in der Diagnostik und Therapie der Uveitis verfügen. Eine Orientierungshilfe bietet hierbei der von der Deutschen Ophthalmologischen Gesellschaft (DOG) initiierte Qualifikationskurs der Sektion Uveitis, der die standardisierte Fortbildung zur Uveitis fördern soll. Die Vernetzung mit Kolleginnen und Kollegen, die diesen Qualifikationskurs absolviert haben oder in enger Kooperation mit einem Uveitiszentrum arbeiten, kann insbesondere bei Wohnortwechseln während der Transition eine kompetente Betreuung von Patienten mit chronischer JIAU sichern.

Transition setzt eine enge interdisziplinäre Zusammenarbeit voraus

Transition ins Erwachsenenalter ist also ein versorgungsrelevantes Thema. Dies setzt einen strukturierten Transfer aller relevanten Informationen hinsichtlich der Gelenk- und Augenerkrankung voraus. Wichtige Grundvoraussetzung ist eine enge interdisziplinäre Zusammenarbeit. Die vom artikulären Verlauf abhängige mögliche Reklassifikation bietet ggf. auch die Chance, dass für das Uveitismanagement weitere für diese Indikation zugelassene Arzneimittel verfügbar werden. Um die interdisziplinäre Kommunikation zu erleichtern, sollten im Arztbrief des von Ophthalmologie und (Kinder‑)Rheumatologie wichtige Informationen enthalten sein (siehe AWMF-Leitlinie Registernummer 045-012; Tab. 3), welche ein zentrales Element des Transitionsprozesses darstellen.

**Tab. 3:** Informationsaustausch zwischen den pädiatrischen und adulten Behandlungsteams

Rheumatologie	Ophthalmologie
Diagnose und Begleiterkrankungen	Alter bei Uveitisdiagnose
Klassifikation der JIA (ILAR-Kriterien)	Klassifikation der Uveitis (SUN-Kriterien)
Bisheriger Verlauf der artikulären Entzündung	Bisheriger Verlauf der okulären Entzündung
Verlauf unter der bisherigen Therapie	
Aktuelle Therapie	
Unerwünschte Wirkungen unter Therapie	
Aktuelle klinische, laborparametrische und bildgebende Befunde	Visusverlauf
Einschätzung der arzt- und patientengewichteten Krankheitsaktivität (JADAS-Score)	Zellgrad in der Vorderkammer
Patientengewichtete Outcomes (Funktionsfähigkeit im Alltag mit CHAQ, Schmerz und Morgensteifigkeit)	Tensio
Komplikationen der Gelenke/weiterer muskuloskeletaler Strukturen	Strukturelle Komplikationen im Auge
Bisherige Operationen an Gelenken	Bisherige Augenoperationen
Krankenkassenzusagen bisheriger Off-label-Therapien	

*ILAR* International League of Associations for Rheumatology, *SUN* Standardization of Uveitis Nomenclature, *JADAS* Juvenile Arthritis Disease Activity Score, *CHAQ* Childhood Health Assessment Questionnaire

## Therapie der Uveitis im Erwachsenenalter: Was ändert sich – was bleibt gleich?

Wie auch im Kindesalter, werden zur Behandlung der JIAU initial topische Steroide verwendet. Bei persistierender Uveitisaktivität, zu hoher topischer Steroiddosis (> 2-mal/Tag) oder neuen entzündungsbedingten Komplikationen der Uveitis soll eine csDMARD-Therapie (bevorzugt Methotrexat [MTX]) erfolgen. Adalimumab stellt in Kombination mit MTX die Standardtherapie für Patienten mit aktiver, nichtinfektiöser JIAU dar, wenn eine alleinige MTX-Therapie nicht ausreicht. Für die anteriore nichtinfektiöse Uveitis besteht eine Zulassung für die Therapie im Kindes- und Jugendalter [39]. Mit Erreichen des Erwachsenenalters entfällt jedoch die arzneimittelrechtliche Zulassung für die anteriore Uveitis. Dies gilt auch, wenn die Erkrankung vorher schon bekannt und die Therapie bisher erfolgreich war und weiterhin erforderlich bleibt. Im Gegensatz dazu besteht eine Zulassung zur Behandlung einer refraktären nichtinfektiösen Uveitis intermedia, Uveitis posterior und Panuveitis [40]. Die Adalimumab-Behandlung von JIAU-Betroffenen mit Eintritt in das Erwachsenenalter ist daher aus ophthalmologischer Sicht nur in Off-label-Anwendung möglich. Hier ist eine interdisziplinäre Abstimmung mit der Rheumatologie erforderlich, da durch eine bestehende artikuläre Diagnose ein *in-label use* begründet werden kann. Von einer etwaigen Änderung der DMARD hin zu einer Medikation, welche für die entzündlich-rheumatische Grunderkrankung zugelassen ist, kann der Uveitisverlauf sehr profitieren.

Auch in der DMARD-Deeskalation bedarf es der interdisziplinären Zusammenarbeit. Bei anhaltender Inaktivität von Arthritis und Uveitis kann dieser Therapieschritt interdisziplinär erwogen werden. Die Länge der dafür zugrunde liegenden Entzündungsfreiheit ist nach aktueller Studiensituation allerdings unklar [41]. Eine enge interdisziplinäre Therapieabstimmung ist für ein optimales Uveitismanagement daher unerlässlich.

## Fazit für die Praxis

- Die mit einer juvenilen idiopathischen Arthritis (JIA) assoziierte Uveitis kann trotz rückläufiger Gelenkaktivität bis ins Erwachsenenalter persistieren und aufgrund ihres häufig asymptomatischen Verlaufs unbemerkt zu schweren okulären Komplikationen führen.
- Daher sind auch nach Erreichen der Volljährigkeit regelmäßige ophthalmologische Kontrollen und eine strukturierte Transition erforderlich.
- Eine enge interdisziplinäre Zusammenarbeit zwischen Ophthalmologie sowie pädiatrischer und internistischer Rheumatologie ist entscheidend, um Therapieentscheidungen und eine mögliche Deeskalation der krankheitsmodifizierenden Antirheumatika (DMARD) sicher abzustimmen.
- Besondere Aufmerksamkeit erfordern dabei Versorgungslücken und die teilweise fehlende arzneimittelrechtliche Zulassung etablierter Therapien im Erwachsenenalter.

## Data Availability

Alle dieser Arbeit zugrunde liegenden Daten sind in diesem Artikel enthalten.
